# Characterization of the complete chloroplast genome of *Scrophularia cephalantha* endemic to Korea

**DOI:** 10.1080/23802359.2021.1925986

**Published:** 2021-10-12

**Authors:** Hyun-Do Jang, Gi-Heum Nam, Min-Su Park, Jumin Jun

**Affiliations:** a Plant Resources Division, National Institute of Biological Resources, Incheon, South Korea; b Department of Biology Education, Kongju National University, Gongju, South Korea; c Biological Resources and Utilization Division, National Institute of Biological Resources, Incheon, South Korea

**Keywords:** *Scrophularia cephalantha*, Korean endemic plants, Scrophulariaceae, complete chloroplast genome

## Abstract

*Scrophularia* species are highly valued and widely used traditional medicinal plants in East Asia. In this article, the complete chloroplast genome of *Scrophularia cephalantha*, a species endemic to South Korea, is reported for the first time. The genome is 153,016 bp long, and it is composed of a pair of 25,485 bp inverted repeats (IRs), separated by a large single copy (LSC) region of 84,124 bp, and a small single copy (SSC) region of 17,922 bp. There are 133 predicted genes in the genome, comprising 88 protein-coding genes, 37 tRNAs, and 8 rRNAs, with an overall GC content of 38%. Phylogenetic analysis based on the chloroplast genome data showed that *S. cephalantha* is a sister species to *S. buergeriana* and *S. ningpoensis*. The data provide useful molecular information for phylogenetic and evolutionary studies of the genus *Scrophularia* and its related species.

The genus *Scrophularia* L. is one of the largest genera in the Scrophulariaceae and it consists of approximately 200 species mostly distributed in temperate regions of the Northern Hemisphere, with a few species distributed in the Southern Hemisphere (Stiefelhagen 1910; Hong 1983; Hong et al. 1998). *Scrophularia* species are popular traditional medicinal plants in East Asia used to treat inflammation, high blood pressure, and fever (Hwang et al. 1998; National Herbal Medicine Information 2021). Six species are recognized in South Korea, three of which, *Scrophularia cephalantha*, *Scrophularia. koraiensis*, and *Scrophularia takesimensis*, are endemic to the country (Jang and Oh 2013).

*S. cephalantha* Nakai is remarkably well distinguished from its related species, *S. kakudensis* Franch. and *S. koraiensis* Nakai, by broadly ovate-shaped leaves, earlier flowering season, and a lower number of flowers on shorter inflorescences (Jang and Oh 2013). This endemic plant is distributed only in the Nakdong Mountains from Gangwon-do to Gyeongsangnam-do in South Korea (Jang 2016). Therefore, it is necessary to establish a strategy for the conservation of this species. However, the biological and genetic information of the species is lacking. In this study, we sequenced the chloroplast genome of *S. cephalantha* for the first time.

The specimen of *S. cephalantha* was collected from Mireuksan Mt., Tongyeong-si, Gyeongsangnam-do, Korea (34°48′49″N, 128°24′58″E, 249 m a.s.l.), and deposited at the National Science Museum (Seongjin Ji, jsj76@korea.kr) in Daejeon, South Korea under the voucher number H. D. Jang 504. Genomic DNA was extracted using the Exgene^™^ Plant kit (GeneAll, Seoul, Republic of Korea) following the manufacturer’s protocol. Genomic DNA libraries were constructed using the QIAseq^™^ Ultralow Input Library kit (QIAGEN, Hilden, Germany). DNA shearing was performed using Q-Sonica 800 (QSonica, Newtown, CT) following the manufacturer’s protocol for obtaining ∼500 bp fragments. DNA was fragmented using the following enzymatic reaction: blunt-ends were repaired, 3′-adenylated, and ligated with multiplex compatible adapters to construct an Illumina-compatible DNA library. Size selection of the 350–650 bp DNA fragments was carried out using Agencourt AMPure XP SPRI beads. PCR enrichment selectively amplified the DNA containing adapters at both ends. Library validation was performed using a Labchip GX (PerkinElmer, Waltham, Massachusetts) and then quantified using the PicoGreen^™^ dsDNA HS Assay Kit and the KAPA qPCR kit for the library. High-throughput sequencing was performed using the Illumina HiSeq 2500 platform (Illumina Inc., San Diego, CA) at the Genome Analysis Center of National Instrumentation Center for Environmental Management (NICEM, Seoul, Republic of Korea). The run mode was a Rapid Pair End with 250 cycles. We obtained genomic DNA NGS sequences of approximately 10 Gb. *De novo* assembly was conducted using CLC Genomic Workbench version 10.0.0.1 (CLCBio, Aarhus, Denmark https://www.qiagenbioinformatics.com/), and chloroplast genome annotations were performed using Glimmer (http://ccb.jhu.edu/software/glimmer/index.shtml). The chloroplast genome sequence was deposited in GenBank under accession number MN255822.

The complete chloroplast genome of *S. cephalantha* had a typical quadripartite structure with a size of 153,016 bp. This was composed of a small single copy (SSC) region of 17,922 bp, a large single copy (LSC) region of 84,124 bp, and a pair of inverted repeat (IR) regions of 25,485 bp. The GC content of the genome was 38%. The IR regions had a higher GC content (43.2%) compared with that of the LSC (36.1%), and SSC (32.2%) regions. There were a total of 133 genes, and they included 88 protein-coding genes, 37 tRNAs, and 8 rRNAs in the genome. Twenty-one genes were duplicated in the IR regions, of which eight were protein-coding genes (*ndh*B, *rpl*2, *rpl*23, *rps*12, *rps*7, *ycf*1, *ycf*2, and *ycf*15), five were tRNA genes (*trn*A-UGC, *trn*L-CAA, *trn*N-GUU, *trn*R-ACG, and *trn*V-GAC), and four were rRNA genes (*rrn*16S, *rrn*23S, *rrn*4.5S, and *rrn*5S).

To identify the phylogenetic position of *S. cephalantha*, we generated a maximum likelihood tree of seven Scrophulariaceae species based on their complete chloroplast genomes (Figure 1). The sequences were aligned using Geneious Prime version 2019.2.3 (https://www.geneious.com, Auckland, Newzealand). Phylogenetic analyses were performed using the CIPRES Science Gateway webserver (RAxML-HPC on XSEDE version 8.2.10) with 1000 bootstrap replicates (Stamatakis 2014). The phylogenetic tree supported the tribe classification of Srophulariaceae reported in previous studies (Olmstead et al. 2001; Tank et al. 2006). The phylogenetic analysis placed *S. cephalantha* closely related to *S. buergeriana* and *S. ningpoensis* from the series *Kakudenses* (Yamazaki 1949; Jang 2016), with 100% bootstrap support.

**Figure 1.**
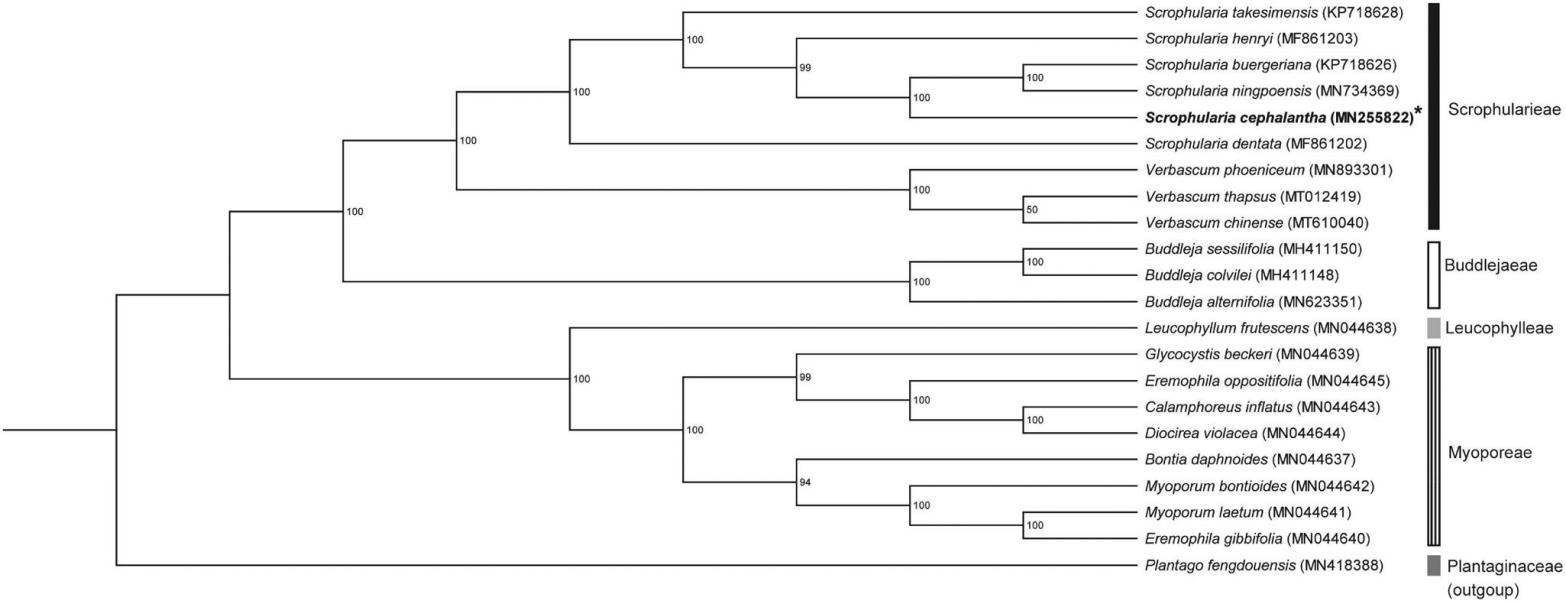
The maximum likelihood tree of family Scrophulariaceae with the outgroup, Plantago fengdouensis, was constructed based on chloroplast genome sequences. Number on the nodes are bootstrap values from 1,000 replicates.

## Data Availability

The genome sequence data that support the findings of this study are openly available in GenBank of NCBI at https://www.ncbi.nlm.nih.gov/ under the accession no. MN255822. The associated BioProject, SRA, and Bio-Sample numbers are PRJNA718412, SRX10472095, and SAMN18534161, respectively.
